# Comparison of Standard 1.5 T *vs.* 3 T Optimized Protocols in Patients Treated with Glatiramer Acetate. A Serial MRI Pilot Study

**DOI:** 10.3390/ijms13055659

**Published:** 2012-05-10

**Authors:** Robert Zivadinov, David Hojnacki, Sara Hussein, Niels Bergsland, Ellen Carl, Jacqueline Durfee, Michael G. Dwyer, Cheryl Kennedy, Bianca Weinstock-Guttman

**Affiliations:** 1 Buffalo Neuroimaging Analysis Center, State University of New York, Buffalo, NY 14203, USA; E-Mails: sarahussein@hotmail.com (S.H.); npbergsland@bnac.net (N.B.); ecarl@bnac.net (E.C.); jdurfee@bnac.net (J.D.); mgdwyer@bnac.net (M.G.D.); ckennedy@bnac.net (C.K.); 2 The Jacobs Neurological Institute, Department of Neurology, University at Buffalo, State University of New York, Buffalo, NY 14203, USA; E-Mails: hojnacki@buffalo.edu (D.H.); BWeinstock-Guttman@kaleidahealth.org (B.W.-G.)

**Keywords:** glatiramer acetate, gadolinium-enhancing lesions, magnetic resonance imaging, 1.5 T, 3 T, T2 lesions, pilot trial

## Abstract

This study explored the effect of glatiramer acetate (GA, 20 mg) on lesion activity using the 1.5 T standard MRI protocol (single dose gadolinium [Gd] and 5-min delay) or optimized 3 T protocol (triple dose of Gd, 20-min delay and application of an off-resonance saturated magnetization transfer pulse). A 15-month, phase IV, open-label, single-blinded, prospective, observational study included 12 patients with relapsing-remitting multiple sclerosis who underwent serial MRI scans (Days −45, −20, 0; the minus ign indicates the number of days before GA treatment; and on Days 30, 60, 90, 120, 150, 180, 270 and 360 during GA treatment) on 1.5 T and 3 T protocols. Cumulative number and volume of Gd enhancing (Gd-E) and T2 lesions were calculated. At Days −45 and 0, there were higher number (*p* < 0.01) and volume (*p* < 0.05) of Gd-E lesions on 3 T optimized compared to 1.5 T standard protocol. However, at 180 and 360 days of the study, no significant differences in total and cumulative number of new Gd-E and T 2 lesions were found between the two protocols. Compared to pre-treatment period, at Days 180 and 360 a significantly greater decrease in the cumulative number of Gd-E lesions (*p* = 0.03 and 0.021, respectively) was found using the 3 T *vs*. the 1.5 T protocol (*p* = NS for both time points). This MRI mechanistic study suggests that GA may exert a greater effect on decreasing lesion activity as measured on 3 T optimized compared to 1.5 T standard protocol.

## 1. Introduction

Gadolinium (Gd) enhancement in multiple sclerosis (MS) lesions correlates with histopathologic findings of blood-brain barrier (BBB) breakdown and active inflammation. [1–4] Gd-enhancing (Gd-E) lesions on post-contrast T 1-weighted images (WI) usually correspond to areas of high signal intensity on T2-WI and low signal intensity on unenhanced T1-WI, probably due to edema and demyelination [5]. Gd-enhancement is a transient phenomenon in MS that usually disappears after several weeks [6]. The presence of continuing enhancement indicates a higher risk of relapses over the short-to-intermediate term and may contribute to long term clinical dysfunction [5]. Various strategies have been proposed to increase the sensitivity of Gd-enhanced MRI for the detection of active MS lesions. These strategies, which maximize the information that can be obtained by Gd-enhancement, include [2]: (1) frequent serial monthly scanning; (2) a delay longer than 5 min between Gd injection and scanning; (3) using doses of higher contrast (e.g., a double or triple dose instead of a standard 0.1 mmoL/kg dose); (4) acquiring thinner tomographic slices; (5) coregistration; (6) reducing the background signal by the application of off-resonance saturation magnetization transfer (MT) pulses to T1-WI and (7) use of high-field strength scanners [7–9].

A number of studies used the standard protocol at 1.5 T (a single dose of Gd, with a 5-min scanning time delay after Gd injection) to compare with the optimized protocols at 1.5 T or 3 T (a triple dose of Gd, with a 20-min scanning time delay after Gd injection, and applied an off-resonance saturated magnetization transfer [MT] pulse) [7,10–13]. These investigations confirmed that use of optimized protocols and/or stronger field strength scanners may increase the ability to detect Gd-E lesions in patients with MS [7–11,13,14]. Gd-E lesions visualized after application of a triple dose of Gd are characterized by a smaller increase in BBB permeability and milder tissue damage than those enhancing after a single dose [2].

Interferon-beta (IFN-β) treatment has a robust effect on visible inflammation with the 1.5 T standard protocol (when BBB is largely disrupted). This is one of the principal reasons why a decrease of >80% is observed in macroscopically visible Gd-E lesions with IFN-β treatment [15]. A previous study showed that IFN-β-1a subcutaneously (S.C.) (44 μg) had a significantly greater effect on reducing the number of Gd-E lesions visible with a triple dose of Gd than on reducing the number of Gd-E lesions visible with a single dose of Gd on the 1.5 T scanner [16]. Another study found that, compared to the effect of IFN-β-1a S.C. (44 μg) given once a week, the decrease in the appearance rate of new Gd-E enhancing lesions was better with a triple dose of Gd than with a single dose of Gd using a 1.5 T scanner [17]. These findings fit with the concept that IFN-β affects the BBB permeability in situ, either directly or by decreasing the release of proinflammatory cytokines [18]. On the other hand, a study using single and triple doses of Gd on 1.5 T scanner in MS patients treated with glatiramer acetate (GA) S.C. (20 mg) showed that GA reduced equally the mean numbers of Gd-E lesions evidenced on both single and triple doses of Gd, without interaction with the dose of Gd [19].

More recently, in the BECOME (Betaseron *vs.* Copaxone in MS with Triple-Dose Gadolinium and 3-T MRI Endpoints) study, the effect of IFN-β-1b and GA on new active lesions was tested on the 3 T optimized protocol (3 T MRI scanner, a triple dose of Gd, with a 20-min scanning time delay after Gd injection) in 75 patients with relapsing-remitting (RR) MS who underwent serial monthly MRI scans over 12 months [20]. No differences in treatment effect on new active or Gd-E lesions were seen between the two treatment arms. However, in two larger head-to-head studies that evaluated clinical and MRI differences between GA and high-dose IFN-β on standard 1.5 T MRI with a single dose of Gd, advantages in suppressing Gd-E lesions were observed with high-dose IFN-β [21,22]. In the REGARD (Rebif *vs.* Glatiramer Acetate in Relapsing MS Disease) study, MS patients treated with IFNβ-1a S.C. (44 μg) had significantly fewer Gd-E lesions (0.24 *vs*. 0.41 per patient per scan, *p* = 0.0002), than those treated with GA [21]. In the BEYOND (Betaferon/Betaseron Efficacy Yielding Outcomes of a New Dose in Multiple Sclerosis [MS] Patients) study, which compared 2 doses of IFNβ-1b S.C. (250 μg or 500 μg) with 20 mg GA daily in 2244 patients, the cumulative volume, but not the cumulative number, of Gd-E lesions from baseline to last available scan was significantly lower (*p* = 0.028) for 500 μg IFNβ-1b (non-marketed dose) compared with GA [22]. All in all, data from these recent head-to head trials that used 3 T optimized and 1.5 T standard protocols suggest that GA may favorably affect early events in lesion formation (smaller lesions visible only on 3 T optimized protocol), in addition to exerting more transient beneficial effects on established areas of inflammation and demyelination (larger lesions visible on 1.5 T standard protocol). Furthermore, GA may have a more diffuse, less focal action in the brain. This action can be recognized only on the 3 T optimized protocol [23].

Based on this background, we hypothesized that use of the 3 T optimized compared to 1.5 T standard protocol may shed light on GA’s mechanism of action. Therefore, the aim of this pilot study was to investigate mechanisms by which GA influences inflammation in MS as evidenced by serial MRIs. Specifically, the present study had 2 objectives: (1) to explore whether the 3 T optimized *vs*. the 1.5 T standard protocol provides stronger evidence that GA decreases inflammation as shown by a decrease in the cumulative number of Gd-E lesions over 180 and 360 treatment days, and (2) to compare whether the decrease in the cumulative number of Gd-E lesions significantly differs between pre-treatment and post-treatment (180 and 360 days) periods using 1.5 T standard and 3 T optimized protocols.

## 2. Results

Of the 12 RRMS patients enrolled, 8 patients completed the 180- and 360-day clinical and MRI follow-up. Of the remaining 4 patients, 1 dropped out at Day 60 due to pregnancy; 1 left the study at Day 90 due to implantation of a metal device incompatible with MRI scanning; 1 dropped out at Day 120 due to an inability to adhere to the MRI schedule; and 1 discontinued the study at Day 150 due to incarceration.

The clinical characteristics of all enrolled patients (Day −45) and those who completed MRI assessments at Days 180 and 360 (completers) are provided in Table 1. There were no statistically significant differences for clinical and demographic characteristics between enrolled patients and completers.

In terms of clinical results, 7 relapses occurred during the year prior to study treatment initiation and 4 of those were recorded in the pre-treatment period; 6 relapses occurred during the 180 treatment period; no relapses occurred in 180–360 treatment period. All relapses were treated with 1 g MP by IV infusion daily for 3–5 days. Overall, 3 (25%) of the 12 patients presented with a relapse during the treatment period. EDSS scores remained stable, albeit slightly improved, during the 180 and 360 days of treatment, with mean scores of 2.8 ± 0.8 and 2.7 ± 0.6, respectively. No serious adverse events (AEs) were recorded. Patients showed the usual AEs as indicated in the package insert for GA, but AEs were not systematically recorded.

MRI characteristics prior to GA treatment at Days −45 and Day 0 for the 1.5 T standard and 3 T optimized protocols are shown in Table 2. The mean number of Gd-E lesions was significantly lower using the 1.5 T *vs*. 3 T protocol at Day −45 (*p* = 0.01) and Day 0 (*p* = 0.0006). The mean Gd-E lesion volume at Day −45 and Day 0 using the 1.5 T was significantly lower than that obtained with the 3 T optimized protocol (*p* < 0.0001 and *p* = 0.04, respectively). Mean T1 and T2 lesion numbers and volumes at Day −45 and Day 0 were also calculated, but differences did not reach statistical significance.

**Figure 1:**
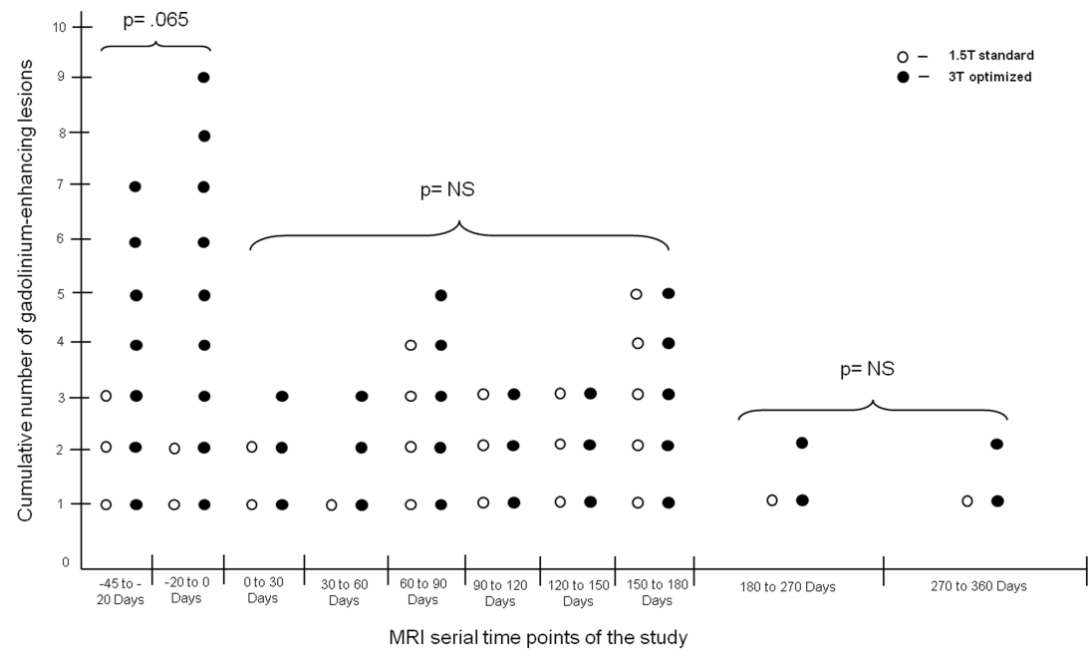
Differences in cumulative number of gadolinium-enhancing lesions between 1.5 T standard and 3 T optimized protocols on serial MRI time points of the study.

Table 3 and Figure 1 show the cumulative lesion activity and number of active scans (defined as a scan with a Gd-E lesion) evidenced during the pre-treatment period (Days −45 to Day 0), treatment period (Days 0–180 and Days 180–360) using the 1.5 T standard and 3 T optimized protocols. There was a trend for lower mean cumulative number of Gd-E lesions during pre-treatment using the 1.5 T standard versus the 3 T optimized protocol (2.5 ± 2.1 *vs*. 5.3 ± 5.9; *p* = 0.065). However, at 180 and 360 days, there was no difference in the mean cumulative number of Gd-E lesions using the 1.5 T *vs*. the 3 T protocol.

During the pre-treatment period, 6 patients (50%) had active Gd-E scans using the 1.5 T standard protocol and 9 patients (75%) had active Gd-E scan using the 3 T optimized protocol (*p* = NS). However, at Days 180 and 360 during treatment, the number of patients having active Gd-E scans was 4 (33.3%) for 1.5 T and 3 T; *p* = NS.

During the 180 and 360 treatment periods of the study, there were no differences in the cumulative number of new Gd-E and T2 lesions using the 3 T optimized and the 1.5 T standard protocol, although there was significantly higher Gd-E lesion activity at Day 0 on the 3 T optimized compared with the 1.5 T standard protocol (*p* = 0.0006). Compared to the pre-treatment period, at Days 180 and 360, a significantly greater decrease in the cumulative number of Gd-E lesions (*p* = 0.03 and 0.021, respectively) was found using the 3 T *vs*. the 1.5 T protocol (*p* = NS for both time points).

The mean cumulative T2 lesion number was approximately half using the 1.5 T standard (1 ± 1) compared with the 3 T optimized protocol (2.5 ± 2.1; *p* = NS) during pre-treatment, but there was a similar cumulative number of T2 lesions at Days 180 and 360 during treatment. The number of patients having active T2 scans during pre-treatment was 4 (33.3%) using the 1.5 T standard protocol, and 6 (50%) using the 3 T optimized protocol (*p* = NS). As with the number of active Gd-E scans per patient, the number of patients with active T2 scans was non-significant regardless of protocol type (1.5 T or 3 T) or time period (180 or 360 days) during treatment (3 patients).

## 3. Discussion

This was a pilot mechanistic study, evaluating the evolution of Gd-E lesions in patients with RRMS through serial scans using 1.5 T standard and 3 T optimized protocols. During the 180- and 360-day treatment periods, there were no significant differences in the cumulative number of Gd-E and T2 lesions using the 1.5 T standard *vs*. the 3 T optimized protocol, although there was significantly higher Gd-E lesion activity at Day 0 on the 3 T optimized versus the 1.5 T standard protocol and a trend for higher cumulative Gd-E lesion number in the pre-treatment period. When compared with the pre-treatment period, there was a significant decrease in the mean cumulative number of Gd-E lesions in the treatment period with the 3 T optimized but not with the 1.5 T standard protocol. These MRI findings suggest that GA exerted a more beneficial effect on Gd-E on the 3 T optimized compared with the 1.5 T standard protocol by probably inhibiting early events in lesion formation. Therefore, GA may favorably affect early events in lesion formation, in addition to exerting more transient beneficial effects on established areas of inflammation and demyelination. In other words, GA can exert an additional effect on smaller lesions not macroscopically visible on a 1.5 T standard protocol.

A single dose of Gd 1.5 T standard MRI provides only indirect information on the degree of inflammation that accompanies active MS lesions, and does not take into account the presence of smaller Gd lesions in the normal appearing (NA) white matter (WM) and gray matter (GM) [2]. However, Gd lesions enhancing only after a triple dose on 3 T MRI are characterized by a smaller increase in BBB permeability and milder tissue damage than those enhancing after a single dose of Gd. Therefore, the use of standard *vs*. optimized protocols may be appropriate for investigating the treatment effect on events responsible for early microscopic pre-enhancing lesion formation in the NAWM and NAGM that occur in the absence of or during partial BBB breakdown [24].

The results from this study confirm results of the previous study by Rovaris *et al.* [19], which examined the effect of GA on MS lesions enhancing at different Gd doses. The data from this study revealed that GA significantly reduces the mean numbers of Gd-E lesions per patient per month, using standard and triple doses of Gd independent of the severity of the 1.5 T MRI-detectable inflammatory process capable of being tracked via different doses of Gd. It was hypothesized that the absence of a significant interaction between the Gd dose and treatment efficacy was perhaps due to a homogeneous effect of GA on Gd-E lesions that may not be dependent on the severity of BBB disruption [19]. Activation of blood-borne effector mononuclear cells that determine BBB disruption and Gd enhancement is driven primarily by antigen-specific T cells [19]. Rovaris *et al.*, therefore, suggested that GA may act via inhibition of antigen-specific T cells rather than directly on effector cells, which may be the mechanism by which GA affects early events in lesion formation, in addition to exerting more transient beneficial effects on established areas of inflammation and demyelination. Because GA was equally effective on single and triple Gd dose 1.5 T scans in the previous study [19] and on 1.5 T standard *vs*. 3 T optimized protocol in the current study, it could be hypothesized that GA can influence the inflammatory events responsible for early microscopic pre-enhancing lesion formation in the NAWM and NAGM that occur in the absence of or during partial BBB breakdown. The effect of GA on slowing deterioration with non-conventional MRI measures was previously investigated [23,25,26].

Although still not fully elucidated, several mechanisms of action (MOA) have been proposed for GA (generating suppressor cells, inducing tolerance, expanding regulatory T-cell populations, and altering antigen-presenting cells) [27]. Contrary to the MOA of IFN-β [28], the anti-inflammatory activity of GA does not require a blockade of the BBB; rather, GA-specific T-cells are believed to enter the central nervous system to exert anti-inflammatory effects [27,28]. Surrogate biomarkers of MS disease activity are being sought to assess the efficacy of various therapeutic interventions in individual patients, as well as in clinical trials. Use of 1.5 T standard *vs*. 3 T optimized protocols offer the potential to provide this information.

The hypothesis that GA administration might inhibit formation of smaller areas of demyelination (not visible with either a single or triple dose of Gd on the 1.5 T scanner) has been advanced with the results of the BECOME study [20]. There was a similar median (75th percentile) number of combined active lesions per patient per scan for months 1–12: 0.63 (2.76) for IFNβ-1b and 0.58 (2.45) for GA (*p* = 0.58). The primary outcome of the study after 12 months did not distinguish between the two treatment arms, although patients on GA exhibited 441 Gd-E lesions and patients on IFNβ-1b developed a total of 913 (472 more than in the GA arm) [20]. The BECOME study’s findings suggest that IFN-β and GA may facilitate repair in the majority of new brain lesions. Also, GA may have decreased the number of smaller Gd-E lesions visible on the 3 T optimized protocol that were probably not visible on the 1.5 T single- or triple-dose Gd protocols. Future studies using emerging disease-modifying treatments may benefit from using enhanced MRI protocols.

This pilot study had potential limitations that may influence interpretation of our findings, such as a small study population and absence of a control group. The study was designed as a proof-of-concept pilot investigation, to provide sufficient evidence which could justify additional longitudinal studies with a larger sample size to examine head-to-head disease-modifying agent differences on standard and optimized MRI protocols. Based on the current results, an active control group, to be compared with GA, is recommended for future studies using a similar study design. The serial monthly MRI scanning on both 1.5 T and 3 T MRI scanners (>200 MRI scans in 12 MS patients over 15 months) was one of the main limits in successful recruiting of larger sample size of MS patients. However, a careful randomization and blinding of the MRI scanner protocols at every time point of the study strengthens the validity of our study results. Although the regression to the mean could have affected frequency of Gd-E lesions over time, this effect should have been equally distributed between both standard and optimized MRI protocols. In addition, the drop-out rate was relatively high in the present study; however, those patients who stopped the study drug were asked to remain in the study and were included in the ITT analysis, where the month of the drop-out was added to the analyses as a covariate. In order to ensure that the presence of limited time points for 4 patients who dropped-out from the study did not skew the analyses, the analyses were repeated without including the 4 patients. The results were similar to the IIT findings, and therefore it was decided that the IIT results should be presented.

## 4. Materials and Methods

### 4.1. Patient Population

The study population included 12 consecutively enrolled patients with RRMS who satisfied inclusion and exclusion criteria. Briefly, patients were 18 to 65 years of age, had disease duration of 3 months to 20 years, and were diagnosed with RRMS according to the McDonald criteria [29]. Patients had to have 1 Gd-E lesion within 30 days on a screening scan and/or an acute relapse within 3 months prior to study screening. Kurtzke Expanded Disability Status Scale (EDSS) scores had to be ≤5.5 [30]. Although treatment with steroids was allowed for relapses prior to study randomization, patients were excluded if they had received immunomodulatory or immunosuppressant treatment (with [IFN-β, GA, mitoxantrone, cyclophosphamide, cladribine, fludarabine, cyclosporine, total body irradiation, azathioprine, methotrexate, intravenous [IV] immunoglobulin, mycophenolate mofetil, or natalizumab) during the 30 days prior to Day −45 of the study. Patients were also excluded if they had renal disease, due to the potential risk of complications from Gd exposure.

### 4.2. Study Design

This was a 15-month, phase IV, open-label, single-blinded, prospective MRI observational study. All patients were GA-naive and received monotherapy with GA (20 mg/day S.C.) every day beginning at the baseline visit (Day 0) for 12 months; no special precautions were necessary before using GA. IV methylprednisolone (IVMP) was allowed for relapses (1 g MP by IV infusion daily for 3–5 days). Patients were permitted to use additional medications, such as antidepressants, for symptom control. The study was approved by the local Institutional Review Board and all patients gave informed consent (Clinicaltrials.gov identifier number: NCT00937157).

### 4.3. MRI Methods

#### 4.3.1. MRI Acquisition

All potential patients underwent a screening scan using the 1.5 T standard protocol (to confirm the presence of at least 1 Gd-E lesion). Patients enrolled in the study then received serial MRI scans of the brain with the 1.5 T standard and 3 T optimized protocols at Day−45, Day−20, Day 0, Day 30, Day 60, Day 90, Day 120, Day 150, Day 180, Day 270, and Day 360. The interval between 1.5 T standard and 3 T optimized protocols was at least 72 h to avoid effects from the first Gd injection. The scanner order of 1.5 T standard *versus* 3 T optimized protocol was randomized in blocks of 4. The standard T1-WI post-contrast scan utilized a standard 0.1 mmoL/kg dose of Gd with a 5-min delay, whereas the triple dose 3 T optimized protocol used a 0.3 mmoL/kg dose of Gd with a 20-min delay and application of an MT saturation pulse. All patients underwent MRI scans of the brain on the same 2 scanners: 1.5 T General Electric Signa Excite 4x/Lx, Milwaukee, WI, and 3 T General Electric Signa Excite 4x/Lx, Milwaukee, WI. The scanning protocol on 1.5 T and 3 T scanners included: axial fast-spin echo proton density (FSE) PD/T2, axial spoiled gradient recalled (SPGR), axial pre- and post-contrast T1-WI with and without MT pulse, and axial fluid attenuation inversion recovery (FLAIR).

The standard 1.5 T MRI protocol was applied with a 256 × 192 matrix and a field of view (FOV) of 24 cm. Other parameters included the following: the dual FSE PD/T2 sequence was acquired with echo time (TE) and repetition time (TR) of TE1/TE2/TR = 10/90/7475 ms, echo train length (ETL) of 12, 46 slices, 3 mm thick, no gap, 1 average, and an acquisition time (AT) of 4:07 (min:s); axial SE T1 imaging before and 5 min after contrast injection with TE/TR = 12/450 ms, 46 slices, 3 mm, no gap, 1 average, AT of 4:27; and FLAIR sequence with TE/TI/TR = 120/2000/8000 ms, (TI-inversion time) 46 slices, 3 mm thick, no gap, 1 average, AT = 5:21.

The 3 T optimized MRI protocol was applied with a 256 × 256 matrix and a FOV of 25.6 cm. Other parameters were the following: for the PD/T2 sequence the TE1/TE2/TR = 12/95/3000 ms, ETL = 14, 47 slices, 3 mm, no gap, 1 average, AT = 4:31; axial SE T1 imaging before and 20 min after contrast injection with TE/TR = 9/600 ms, 47 slices, 3 mm, no gap, 1 average, AT = 12:22, an 8 ms Fermi MT pulse was used on the 3 T MRI with a 1200 Hz off-resonance frequency to reduce the background signal, and FLAIR sequence with TE/TI/TR = 120/2100/8500 ms, 46 slices, 3 mm thick, no gap, 1 average, AT = 4:16.

#### 4.3.2. MRI Analysis

MRI investigators were blinded to the field strength/protocol type and to the patients’ clinical characteristics and clinical status.

The number and lesion volume (LV) of Gd-E, T2, and T1 lesions were calculated using a highly reproducible semiautomated technique. [31] Lesion activity analysis included calculation of Gd-E and T2 active lesions and active scans on the 1.5 T standard and 3 T optimized protocols after coregistration of serial time points. Coregistration was performed using the FMRIB’s Linear Image Registration Tool (FLIRT) tool [32] to place all scans for a single patient into the same physical space as the pre-contrast T1-WI from Day −45. All coregistrations were run using six degrees of freedom, a correlation ratio cost function, and trilinear interpolation. Although FLIRT is fully automated, the results were manually checked by an operator to ensure their accuracy.

Gd-E lesion was defined as a well demarcated area of unequivocally increased hyperintense signal intensity on post-contrast T1-WI compared with surrounding normal-appearing brain tissue (NABT) that is not hyperintense on the pre-contrast T1-WI. [33] The hyperintensities were defined as Gd-E lesions if the signal increase was not related to a normally enhancing structure (e.g., a vessel or choroid plexus) and not identified as a flow-related artifact. Small Gd-E lesions had to be present in more than one slice. T2 confirmation was mandatory for difficult areas, such as the posterior fossa (with many flow artifacts and confusing tentorial vessels) and the temporal lobes. The mean cumulative number of Gd-E lesions per patient over the 45-day pre-treatment and 180- and 360-day treatment period was measured.

A new or enlarging T2-WI lesion was defined as a rounded or oval lesion arising from an area previously considered NABT or a lesion showing an identifiable increase in size from a previous time point, respectively. Lesion activity was calculated using a reliable contouring-thresholding technique, as previously reported [31,34]. The mean cumulative number of T2 lesions per patient over the 45-day pre-treatment and 180- and 360-day treatment period were measured.

The percentage of active scans showing one or more new enhancing, or new or newly enlarging T2 lesions was also determined. An active scan was defined as a scan showing any new, enlarging, or recurrent lesion on post-contrast T1- and T2-WI.

### 4.4. MRI, Clinical, and Safety Time Points

The serial MRI scans were acquired in the pre-treatment period: screen, Days −45, −20, and 0, and during the treatment period: Days 30, 60, 90, 120, 150, 180, 270 and 360, using the relevant 1.5 T (standard) and 3 T (optimized) MRI protocols. Three pre-treatment MRI scans were used to calculate the baseline level of activity on the 1.5 T standard and 3 T optimized protocols. After treatment with GA (20 mg/day S.C.) began (Day 0), patients underwent 6 consecutive monthly MRI scans at Days 30, 60, 90, 120, 150 and 180. Additional scans were acquired on Day 270 and Day 360, during GA treatment. In total, patients had 22 MRI scans during the 15 months of the study.

The clinical visits occurred at Days −45, 0, 90, 180, 270 and 360, as well as during unscheduled relapses. Patients were evaluated based on physical examinations, routine laboratory examinations for safety monitoring and pregnancy, as well as neurologic assessments.

### 4.5. Endpoints

The primary endpoint was to determine a decrease in inflammatory activity, as evidenced by a decrease in the number of Gd-E lesions with the 3 T optimized and 1.5 T standard protocols in the treatment period (Days 180 to 360), when compared with the pre-treatment period (Days −45 to 0). The secondary endpoint of the study was to compare the cumulative number of Gd-E lesions acquired with the 3 T optimized and 1.5 T standard protocols during Days 180 and 360.

### 4.6. Statistical Analysis

All data analyses were performed using SPSS version 16.0 (SPSS, Inc., Chicago IL). For comparisons between the groups, the chi square test, the Student’s *t*-test, the Mann-Whitney U test and the Wilcoxon rank sum tests were used, as appropriate.

Because some data were missing for the post-baseline MRI endpoints, the principle of “last observation carried forward” was used for the value. If no post-baseline value was available for a subject, then the mean value was calculated at the specified visit using all available data. The mean value was used as the imputed value.

All 12 enrolled patients entered into the intention-to-treat (ITT) analysis. Patients who stopped the study drug were asked to remain in the study for the ITT analysis, but a covariate for the month of the drop-out was added.

All *p* values were based on two-tailed tests and the minimum significant level was *p* < 0.05 and a trend *p* < 0.1.

## 5. Conclusion

GA may favorably affect early events in lesion formation, in addition to exerting more transient beneficial effects on established areas of inflammation and demyelination, an effect that may be observed only with the 3 T optimized protocol and not the 1.5 T standard protocol.

## Figures and Tables

**Table 1:** Demographic and clinical characteristics of enrolled patients and those completing the study.

Clinical Characteristics	Enrolled Patients (*N* = 12)	Completers (*n* = 8)
% Female	75	83.3
Age (y), mean (SD)	43 (7.8)	43.1 (7.7)
Age onset (y), mean (SD)	31.3 (10.4)	31.2 (10.1)
Disease duration (y), mean (SD)	12.8 (7.9)	12.6 (7.8)
Relapses in 1 year before study entry, mean (SD)	0.92 (0.99)	0.9 (0.8)
Number of relapses in 1 year before study entry, *n* (%)		
0	5 (41.6)	4 (50)
1	4 (33.3)	2 (25)
2	2 (16.6)	1 (12.5)
3	1 (8.3)	1 (12.5)
EDSS, mean (SD) median	2.7 (1.1) 2.5	2.8 (0.8) 2.5

**Legend:** SD = standard deviation; EDSS = Expanded Disability Status Scale. There were no statistically significant differences between patients enrolled in the study and the study completers for baseline clinical and demographic characteristics. Patients who stopped the study drug were asked to remain in the study and were analyzed with the intention-to-treat, but adding a covariate for the month of the drop-out.

**Table 2:** MRI characteristics at Days −45 (*i.e.,* 45 days before treatment started) and Day 0 on 1.5 T standard and 3 T optimized protocols.

	Day −45 1.5 T Standard Protocol (*n* = 12)	Day −45 3 T Optimized Protocol (*n* = 12)	*p* value	Day 0 1.5 T Standard Protocol (*n* = 12)	Day 0 3 T Optimized Protocol (*n* = 12)	*p* value
Gd-E number, mean (SD) min–max	2.7 (2.1) 1–5	6.2 (4.1) 1–11	0.01	0.8 (0.6) 0–3	3.1 (1.9) 1–8	0.0006
Gd-E volume, † mean (SD)	0.33 (0.07)	0.66 (0.2)	<0.0001	0.1 (0.1)	0.36 (0.4)	0.04
T2 lesion number, mean (SD) min-max	37 (37.8) 10–71	42.6 (24.7) 26–93	NS	28.7 (27) 10–83	35.1 (21.4) 12–99	NS
T2 LV, mean (SD)	26.1 (38.1)	34.6 (44.6)	NS	22.2 (25.9)	28.8 (29.1)	NS
T1 lesion number, mean (SD) min-max	11.3 (10) 26–45	25 (17) 44–75	NS	14.9 (15.4) 3–49	21 (15.1) 4–51	NS
T1 LV, mean (SD)	2.4 (3.7)	5.1 (5.6)	NS	2.5 (4.1)	5.8 (5.9)	NS

**Legend:** Gd-E = gadolinium-enhancing lesion; SD = standard deviation; LV = lesion volume; NS = non-significant. All MS patients at screening presented at least 1 Gd-E lesion on the 1.5 T standard protocol. There was a significant difference between 1.5 T standard and 3 T optimized protocols in Gd-E number and volume at Days −45 and Day 0. For comparisons, the Student’s *t*-test and Wilcoxon rank sum test were used.

† The LV results are expressed in milliliters.

**Table 3:** MRI cumulative lesion activity and active scans in the pre-treatment (Days −45 to Day 0) and treatment periods (Days 0–180 and 0–360) on 1.5 T standard and 3 T optimized protocols.

	−45 to 0 Days 1.5 T Standard Protocol (*n* = 12)	−45 to 0 Days 3 T Optimized Protocol (*n* = 12)	*p* value	0–180 Days 1.5 T Standard Protocol (*n* = 12) *	0–180 Days 3 T Optimized Protocol (*n* = 12) *	*p* value	0–360 Days 1.5 T Standard Protocol (*n* = 12) *	0–360 Days 3 T Optimized Protocol (*n* = 12) *	*p* value
Cumulative Gd-E number, mean (SD) min-max (sum)	2.5 (2.1) 1–4 (5)	5.3 (5.9) 1–12 (16)	0.065	3.5 (4.1) 1–10 (18)	3.7 (5.8) 1–15 (22)	NS	3.6 (4.2) 1–11 (20)	3.8 (5.4) 1–15 (24)	NS
Cumulative T2 lesion number, mean (SD) min-max (sum)	1 (1) 0–1 (2)	2.5 (2.1) 1–4 (5)	NS	3.2 (2.6) 2–9 (24)	3.3 (3) 1–9 (30)	NS	5 (3.6) 2–9 (40)	5 (3.3) 1–9 (41)	NS
Active Gd-E scan per patient, number (%)	6 (50)	9 (75)	NS	4 (33.3)	4 (33.3)	NS	4 (33.3)	4 (33.3)	NS
Active T2 scan per patient, number (%)	4 (33.3)	6 (50)	NS	3 (25)	3 (25)	NS	3 (25)	3 (25)	NS

* All available pairs of scans (1.5 T and 3 T) in the pre- and post-treatment periods were used in the analysis. All 12 enrolled patients entered into the intention-to-treat (ITT) analysis. Because some data were missing for the post-baseline MRI endpoints, the principle of “last observation carried forward” was used for the value. If no post-baseline value was available for a subject, then the mean value was calculated at the specified visit using all available data. The mean value was used as the imputed value.

Gd-E = gadolinium-enhancing lesion; SD = standard deviation; NS = non-significant; Cumulative number of Gd-E lesions represents sum of all Gd-E lesions at serial time points. For comparisons, the chi square test, Student’s *t*-test and Wilcoxon rank sum test were used, as appropriate.
